# Pabon Lasso and Data Envelopment Analysis: A Complementary Approach to Hospital Performance Measurement

**DOI:** 10.5539/gjhs.v6n4p107

**Published:** 2014-04-10

**Authors:** Mohammad Mehrtak, Hasan Yusefzadeh, Ebrahim Jaafaripooyan

**Affiliations:** 1 Department of Health Services Management, School of Health Management and Information Sciences, Iran University of Medical Sciences, Tehran, Iran; 2 Department of Health Economics, School of Health Management and Information Sciences, Iran University of Medical Sciences, Tehran, Iran; 3 Health Management and Economics Research Center, School of Health Management and Information Sciences, Iran University of Medical Sciences, Tehran, Iran

**Keywords:** DEA, hospital, Iran, performance measurement, Pabon Lasso model

## Abstract

**Background:**

Performance measurement is essential to the management of health care organizations to which efficiency is per se a vital indicator. Present study accordingly aims to measure the efficiency of hospitals employing two distinct methods.

**Methods:**

Data Envelopment Analysis and Pabon Lasso Model were jointly applied to calculate the efficiency of all general hospitals located in Iranian Eastern Azerbijan Province. Data was collected using hospitals’ monthly performance forms and analyzed and displayed by MS Visio and DEAP software.

**Results:**

In accord with Pabon Lasso model, 44.5% of the hospitals were entirely efficient, whilst DEA revealed 61% to be efficient. As such, 39% of the hospitals, by the Pabon Lasso, were wholly inefficient; based on DEA though; the relevant figure was only 22.2%. Finally, 16.5% of hospitals as calculated by Pabon Lasso and 16.7% by DEA were relatively efficient. DEA appeared to show more hospitals as efficient as opposed to the Pabon Lasso model.

**Conclusion:**

Simultaneous use of two models rendered complementary and corroborative results as both evidently reveal efficient hospitals. However, their results should be compared with prudence. Whilst the Pabon Lasso inefficient zone is fully clear, DEA does not provide such a crystal clear limit for inefficiency.

## 1. Introduction

Measurement is central to quality improvement in organizations (Zhu, 2003), insofar as there exist such popular confirmatory adages as ‘it is impossible to understand what is not measurable and if something cannot be understood, it cannot be improved’ (Halachmi, 2002). Efficiency is further key to the performance measurement as the latter is defined by Neely, Adams and Kennerley (2002) as ‘the process of quantifying the efficiency and effectiveness of past actions’. Efficiency refers to the resource utilization, while effectiveness mostly evaluates the outcomes (Ozcan, 2007). The long-lasting nature of healthcare-related outcomes has de facto made performance measurement challenging and problematic (Eddy, 1998; de Bruijn, 2002), pushing assessors to mostly rely on efficiency- i.e. quantitative- measures. Efficiency is also considered in planning to contain hospitals’ costs, as the latter is swallowing a high proportion of health care funding (Mc Kee & Healy, 2002).

Despite various methods and frameworks of measuring health care organizations’ performance such mainly as balanced scorecard, performance pyramid system, regulatory inspection, third-party assessment and statistical indicators (Shaw, 2003; Kaplan & Norton, 2001; Lynch & Cross, 1991), no consensus upon an appropriate performance measurement approach in health services has emerged (Veillard, Guisset, & Garcia-Barbero, 2004). Therefore, there has been always an endless interest in developing and utilizing a combination of methods and frameworks, as possible, for measuring organizational performance, in a bid to give a rounded picture of organizations’ functionality.

Indices such as bed occupancy rate (BOR^1^), bed turnover rate (BTR^2^) and average length of stay (ALS^3^) represent clear proxies of hospital performance in a quantitative way. Furthermore, hospitals’ capacity utilization rate using their inputs and outputs could be instrumental in estimating efficiency, that is, simply as outputs divided by inputs. Accordingly, the two most common models to measure efficiency which utilize the foregoing indices and indicators are Pabon-Lasso and Data Envelopment Analysis (DEA) (Ajlouni et al., 2013). They both assume a synthetic approach to using hospital indices, inputs and outputs to calculate hospital efficiency. An assessment based on only one of those may be flawed and misleading, whilst utilization of two assessment methods, as the current study aims, in addition to providing a better picture could also render comparable results of hospitals’ efficiency.

The Pabon Lasso model (Bontile, 2013; Kiadaliri, Jafari, & Gerdtham, 2013; Asbu et al., 2012; Ajlouni et al., 2013) and DEA (Kirigia, 2013; Olivares-Tirado & Tamiya, 2014; Ismail, Thorwarth, & Arisha, 2014) have been separately mentioned in health care literature plenty of times. They have been also used by several domestic studies (Gholipour, Delgoshai, Masudi-Asl, Hajinabi, & Iezadi, 2013; Nekoei Moghadam, Rooholamini, Yazdi Feizabadi, & Hooshyar, 2012; Najarzadeh, Torabipoor, Ghasemzadeh, & Salehi, 2012; Bahadori, Sadeghifar, Hamouzadeh, Hakimzadeh, & Nejati, 2011). Despite this extensive application, the joint use of both techniques is rare and only recently is being appreciated in the literature (Ajlouni et al., 2013; Barati Marnani et al., 2012). This study seeks to contribute to this area by applying two methods in a developing country context and further shed some light on the utility of joint application of two distinct assessment frameworks.

Iran, briefly, owns a two-tier health system; at national and provincial level. Ministry of health and medical education (MOHME) is the main authority in the country responsible for health care. It is mainly charged with planning, policy-making, leading, supervising, funding and evaluating health services and medical education in the country (Mohit, 2000). The health care system at provincial level is run by the Universities of Medical Sciences (UMSs). Different types of hospitals provide services at this level, including public (teaching and clinical), private and military hospitals. The majority of the health care services in the country, nevertheless, are provided by the public hospitals.

### 1.1 Pabon Lasso Model

Originally developed by Pabon Lasso in 1986, it is a technique used for interpreting and comparing hospital efficiency utilizing three hospital indices of BOR, BTR and ALS, simultaneously. In its graphical model, BOR is placed on (X) and BTR on (Y) axis. Given a mathematical correlation between these three indices, a line which starts from zero and passes through each point in the graph shows ALS which increases from left to right and top to bottom consistently (Pabón Lasso, 1986). This graph is divided into four zones by two perpendicular lines; one is drawn from average BOR point on (X) axis and the other from average BTR on (Y) axis. Either standard (least acceptable) value of the indices in a given region/country or the average of all hospitals’ related index could be envisaged as a basis for graph subdivisions. Cautions should be yet made while using the mean as the basis for division, as Lasso argues, because such outliers as single-specialty hospitals e.g. psychiatric or gynecologic with overly long and short ALS and BOR might distort and skew the divisions (Pabón Lasso, 1986).

**Figure 1:**
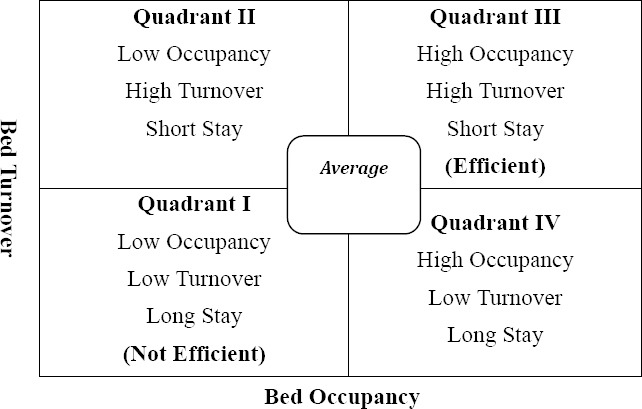
Pabon-Lasso diagram (Pabón Lasso, 1986)

As figure 1 shows the hospitals in the first zone have lower BOR and BTR compared to their mean. There exist more beds with high bed capacity to service-demand ratio and patients seemingly demanding hospitalization are either diverted to other centers or rejected. Hospital development is not required. Physicians are mostly reluctant and demotivated, and the hospitals are not efficient (Sajadi, 2011).

The second zone is characterized by lower BOR and higher BTR than the average. Obstetrics and gynecology hospitals and short-term inpatient centers usually rest in this zone. They have numerous unused and extra beds as well as excessive and hasty hospitalizations. Most of these beds might be used for patients with no need to hospitalization or for their possible examination.

Hospitals in the third zone are both of high occupancy and turnover; pointing to an efficient resource utilization. In fact, a high BTR and BOR indicate that hospitals have reached an appropriate level of efficiency, with relatively few vacant beds at any time. Ultimately, a lower turnover and a higher occupancy than average rate are featured by the quadrant IV. The hospitals in that zone are expected to have long-term inpatients along with less utilization from their resources as well as high costs. More chronic diseases with unnecessary long-term hospitalizations and inefficient service delivery may cause this situation. Psychiatric hospitals and nursing homes are normally put in this area.

The area near the centre is where average hospitals are located (Figure 1). Furthermore, it is important to be noted that plotting the hospitals according to BOR and BTR is only meaningful in the case of hospitals with similar characteristics (eg, only public or private). This classification could assist to understand hospital resource utilization and to recognize facilities that are not used optimally (10), it yet should be interpreted with caution, since variation within the same category of hospitals might twist the results (Ajlouni et al., 2013). Moreover, efficient utilization might not be surely construed as high performance and quality of care.

### 1.2 DEA Technique

Another route to the estimation of hospital efficiency is through examining the relationship between inputs (e.g. resources used in a hospital) and outputs of an organizational process (services delivered) (Kundurjiev & Salchev, 2011). Utilizing these two elements, in the late 70s, DEA, a non-parametric linear programming method, was proposed by Charnes, Cooper and Rhodes (1978) for measuring relative efficiency of non-profit organizations, eg. hospitals. According to DEA, the organizations with a score of 1 (100%) are assumed efficient; whereas the others have efficiency scores of less than 1 (more than zero). Efficient hospitals in a group are seen as best-practice hospitals, role models, and called ‘frontiers’, against which the efficiency of all other hospitals is measured. The following statements bare the virtue of DEA for efficiency measurement in health care (Afzali, Moss, & Mahmood, 2009; Hollingsworth, 2008; Gannon, 2005; Shahhoseini, Tofighi, Jaafaripooyan, & Safiaryan, 2011):

- DEA is able to manage complex service environments (eg. hospitals) which use multiple inputs to deliver various services;
- Efficiency measure in DEA is related to best, not average, practice;
- DEA does not require an explicit relation of inputs and outputs; this is especially relevant in the case of hospitals, as associating their inputs to outputs seems challenging;
- DEA identifies efficient peers for those hospitals that are not efficient. This helps the inefficient hospitals to emulate the functional organization of their peers so as to improve their efficiency;
- DEA can be carried out with either the constant or variable returns to scale (CRS or VRS) assumption (Note 4).

DEA has been de facto developed as a managerial and practical method for measuring hospital performance around the world revealing inefficient and costly areas within the hospitals and assisting to make appropriate decisions for improvement of hospital efficiency (Kengil, Gökmen, & Tozan, 2010). Three types of managerial, technical and scale efficiency (ME, TE, SE) are measureable in DEA (Imami-Meibodi, 2005). TE is the default type meaning that hospitals provide the same level of outputs with lesser amount of resources or higher output with the same level of inputs (Note 5). ME implies that without increasing inputs and only through effective and wise management and effort of employees, efficiency can be boosted. SE as such means that hospitals with IRS should increase their services to act efficiently.

## 2. Method

The present descriptive-analytical study was carried out as cross-sectional and retrospective. Data was collected from all 18 general hospitals of Eastern Azerbaijan (EA) province, the biggest one in the north-west of the country, via Treatment Deputy of EA University of Medical Sciences (EAUMS) using hospitals’ monthly performance forms.

The researchers have to exclude general military and private hospitals owing to data access issues. Furthermore, the specialty hospitals were also ignored given using the mean of indices as the rationale of graph subdivisions. Because, as Lasso explains, single-specialty hospitals e.g. psychiatrics with overly long or short ALS and BOR might skew the divisions (Pabón Lasso, 1986). In fact, since DEA was also being used, requiring that the hospitals be similar, as possible, the general hospitals, located in one province and supervised by a single medical university, were only chosen. As outlier bias was cleared, the hospitals’ mean used for Pabon Lasso graph subdivisions. Given the similar type and structure of all hospitals in the country, this province was mainly chosen on convenience and data access grounds. MS Excel and Visio were employed to display the Pabon Lasso results.

As to the DEA, the accessible inputs for DEA included the number of active beds, physicians, nurses, number of discharged patients from the hospitals (Table 1). Given the sensitivity analysis conducted, only these three outputs had the highest effect on efficiency. Furthermore, the final model was designed based on the variable return to scale (VRS), so as to divide the technical efficiency measure into both managerial and scale efficiency. DEA data was analyzed using DEAP_2.1_ software. Furthermore, in this study, input-oriented TE was addressed, because in health care output maximization as compared to input minimization is mostly out of managers’ control and changing the inputs is much easier and feasible for the managers, than the outputs (Jacobs, Smith, & Street, 2006).

**Table 1:** The hospitals’ inputs and outputs

Hospital	Input				Output		

	Active beds	No. of Physicians	No. of Nurses	Other Professionals	No. of Surgeries	No. of the Discharged	BOR
H1	52	8	28	185	1547	2928	26.1
H2	145	24	80	78	6479	12984	58.8
H3	48	12	22	35	709	1545	16.3
H4	236	17	186	163	10801	12995	66.2
H5	561	7	529	480	10848	25790	72.2
H6	36	5	23	28	1589	3408	44.8
H7	36	16	19	43	873	2068	27.9
H8	106	21	72	130	5899	11342	63.0
H9	119	38	112	156	5628	13283	92.5
H10	144	27	92	99	7767	13466	63.6
H11	50	15	24	30	570	1206	10.1
H12	119	35	96	49	4452	10720	58.1
H13	129	22	68	65	6458	12726	63.5
H14	81	21	53	38	1940	6065	46.7
H15	132	23	82	89	7694	14951	66.0
H16	32	11	32	32	617	2298	43.3
H17	36	15	27	36	943	2252	48.2
H18	72	14	39	45	2209	6090	59.6

## 3. Results

### 3.1 Pabon Lasso Results

**Figure 2:**
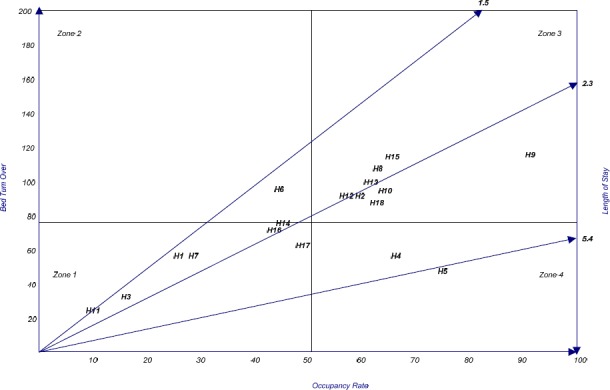
Performance of the hospitals-Pabon Lasso model

Table 2 exhibits the value of given hospitals’ triple indices. The average BOR, BTR and ALS were 51.5%, 76.4 and 2.5%, respectively. Figure 2 also shows that seven out of eighteen hospitals (39%) were in the first zone (having low BOR and BTR, hence inefficient), one (5.5%) in the second zone (with satisfying BTR), eight (44.5%) in the third zone (high BOR and BTR) and, finally, two hospitals (11.1%) were in the fourth zone.

**Table 2:** The hospitals’ indices

Hospital	BTR	ALS	BOR
H9	114.6	2.9	92.5
H8	107.2	2.1	63
H7	57.4	1.8	27.9
H6	94.8	1.7	44.8
H5	49	5.4	72.2
H4	56.5	4.3	66.2
H3	32.2	1.9	16.3
H2	90.2	2.4	58.8
H18	84.9	2.6	59.6
H17	62.6	2.8	48.2
H16	71.9	2.2	43.3
H15	114.6	2.1	66
H14	75.3	2.3	46.7
H13	99.3	2.3	63.5
H12	90.5	2.3	58.1
H11	24.1	1.5	10.1
H10	94.2	2.5	63.6
H1	56.3	1.7	26.1
Mean	76.4	2.5	51.5

### 3.2 DEA Results

As indicated in Table 3, hospital 11 was of the least and hospitals 4,5,9,12,13 and 15 were of the highest efficiency (33.3 percent of the hospitals). Hospital 1 has hence 63 percent slack and needs to reduce its input usage up to this level. Returns to scale for all hospitals is also displayed in the table. Approximately 5.5% of the hospitals showed a DRS, 38.8% with a CRS, and 55.5% had an IRS.

**Table 3:** Hospitals’ efficiency using DEA model

Hospital	SE	ME	TE	Returns to scale
H4	1.000	1.000	1.000	Constant
H5	1.000	1.000	1.000	Constant
H9	1.000	1.000	1.000	Constant
H12	1.000	1.000	1.000	Constant
H13	1.000	1.000	1.000	Constant
H15	1.000	1.000	1.000	Constant
H6	0.983	1.000	0.983	Increasing
H8	0.973	0.981	0.955	Increasing
H10	0.989	0.960	0.949	Decreasing
H18	0.924	1.000	0.924	Increasing
H2	1.000	0.917	0.917	Constant
H14	0.808	0.971	0.785	Increasing
H16	0.635	1.000	0.635	Increasing
H17	0.609	1.000	0.609	Increasing
H1	0.723	0.813	0.588	Increasing
H7	0.582	1.000	0.582	Increasing
H3	0.386	0.972	0.375	Increasing
H11	0.282	0.952	0.269	Increasing
**Mean**	**0.827**	**0.976**	**0.809**	

## 4. Discussion

This study sought to measure the relative efficiency of general public hospitals in an Iranian province. It employed a non-parametric mathematical programming model (DEA) to measure and a graphical analysis (Pabon-Lasso model) to interpret hospital efficiency.

### 4.1 Comparison of DEA and Pabon Lasso Results

As shown in table 4, based on the Pabon Lasso model, 44.5% of the hospitals were entirely efficient (third zone), while DEA revealed 61% to be efficient. As such, 39% of the hospitals, by Pabon Lasso, were utterly inefficient; this was only 22.2% by DEA. Finally, 16.5% of hospitals as calculated by Pabon Lasso (hospitals in the second and fourth zones) and 16.7% by DEA were relatively efficient. Discrepancies are not immense among the results of two methods.

**Table 4:** Comparison of hospitals’ efficiency results

Range of TE score	Explanation	DEA Results	Pabon Lasso Results (Diagram 1)
0.8-1	Efficient	61.1%	44.5%
0.6-0.8	Fairly inefficient	16.7%	16.5%
Less than 0.6	Inefficient	22.2%	39%

Overall, the results implicate that the efficiency status of the hospitals were nearly acceptable as just over half were of the highest range of efficiency when the average of both methods’ results considered (table 4). In fact, approximately 53 percent of the hospitals were fully efficient and 31 percent were fully inefficient. If seen separately, average managerial, technical and scale efficiency for the hospitals were 0.97, 0.80 and 0.82, respectively, implying a favorable performance for the hospitals, though leaving still some space and capacity for improving efficiency in these hospitals (i.e. up to 19.1% TE inefficiency) without any increase in their current expenditure and using the same inputs. Although the average ME demonstrates that management has been outperforming in these hospitals, the percentage of hospitals in the zones 1, 2, 4 (ie. 55.5%) somehow challenges this claim. That is, aligned with Barati and colleagues (2012), DEA revealed more hospitals to be efficient as compared to the Pabon Lasso model.

Most inefficient hospitals (TE<1) were mainly of an IRS, while those efficient of a CRS, signifying that the inefficient hospitals should increase the level of their service delivery and use of full capacity. They de facto suffer from surplus inputs to be reduced, should they wish to reach the highest efficiency (Kirigia, 2013).

Moreover, the findings of both methods also revealed that the larger hospitals do better than smaller ones in terms of efficiency. For example, the large hospitals such as H10, H15 and H13 were more efficient than smaller hospitals (i.e. H17, H16 and H7) in accord with both DEA and Pabon Lasso. This might allude to the fact that hospital size could be a determinant of efficiency, as similarly echoed by Ajlouni and colleagues (2013).

The peer hospitals identified could provide the managers of inefficient hospitals with the best cases they might consider to improve their efficiency and performance, as the peers are not a far cry from inefficient hospitals (Table 5).

**Table 5:** Hospital peers

Hospital	H1	H2	H3	H4	H5	H6	H7	H8	H9	H10	H11	H12	H13	H14	H15	H16	H17	H18
**Hospital peers**	6	15	6	4	5	6	7	15	9	4	7	12	13	6	15	16	17	18
		5	7					9		13	6			12				
		13						6		9				13				
		9								15								
		6																

The peers are in fact those comparatively more efficient hospitals, but similar in terms of their inputs to given inefficient hospital, which could be followed by the latter for efficiency enhancement. As the table displays, the peers perform as a benchmark in a specific order. For instance, the peers for inefficient hospital 8 are hospitals 15, 9 and 6, orderly.

## 5. Conclusion

Performance and efficiency enhancement are among the crucial concerns of policy-makers and managers of health care, nowadays. Hospital indices are assumed as a key proxy for the performance of these organizations. The more the number of indices utilized, the more comprehensive image of performance is expected to materialize. Such measurement methods as Pabon Lasso and DEA at best employ a number of these indices to measure and interpret hospital efficiency. The combinative application of these two methods for a unique purpose further augments this advantage.

Usage of both models provides complementary and corroborative results, but they should be compared with caution. DEA appeared to show more hospitals as efficient as opposed to the Pabon Lasso model. Whilst the Pabon Lasso inefficient zone is entirely clear, DEA does not provide such a crystal clear limit for inefficiency. Nevertheless, both can evidently reveal efficient hospitals. Moreover, both methods can use fairly similar data for assessing performance of the hospitals, which somehow authorizes their comparative use.

Qualitative probing could be a key avenue for further research to uncover the reasons for inefficiencies among the hospitals. Limitations inherent in DEA and Pabon Lasso model should also be noticed. Their deterministic approach and oversimplification, respectively, might to some extent be nonetheless minimized with their complementary application.

## Notes

**Notes**

Note 1. The percentage of beds occupied by patients in a year represented as the ratio of inpatient to active bed-days in a certain period of time.

Note 2. Number of times patients use a bed in a certain period of time achieved through dividing discharges (including the death) in a certain period of time by average active bed at the same period.

Note 3. The average number of days a patient spends in hospital. It is generally measured by dividing the total number of inpatient days during a year by the number of admissions or discharges. Day cases are excluded.

Note 4. The levels of change in output with respect to changes in input level. When by increasing the levels of inputs, more outputs than the proportional rate are produced; it is “increasing returns to scale (IRS)”. In case the output changes by less than the input employed, the ‘decreasing returns to scale (DRS)’.

Note 5. This is the case when ‘efficiency’ term is used.

## References

[ref1] Afzali H. H, Moss J. R, Mahmood M. A (2009). A conceptual framework for selecting the most appropriate variables for measuring hospital efficiency with a focus on Iranian public hospitals. Health Services Management Research.

[ref2] Ajlouni M, Zyoud A, Jaber B, Shaheen H, Al-Natour M, Anshasi R. J (2013). The Relative Efficiency of Jordanian Public Hospitals Using Data Envelopment Analysis and Pabon Lasso Diagram. Global Journal of Business Research.

[ref3] Asbu E, Walker O, Kirigia J, Zawaira F, Magombo F, Zimpita P (2012). Assessing the efficiency of hospitals in Malawi: An application of the Pabón Lasso technique. African Health Monitor.

[ref4] Bahadori B, Sadeghifar J, Hamouzadeh P, Hakimzadeh S, Nejati M (2011). Combining multiple indicators to assess hospital performance in Iran using the Pabon Lasso Model. Australian Medical Journal.

[ref5] Barati Marnani A, Sadeghifar J, Pourmohammadi K, Mostafaei D, Abolhalaj M, Bastani P (2012). Performance assessment indicators: How DEA and Pabon Lasso describe Iranian hospitalsperformance. HealthMED.

[ref6] Bontile H. L. R (2013). Performance of DOH-Retained Hospitals in the Philippines.

[ref7] Charnes A, Cooper W. W, Rhodes E (1978). Measuring the efficiency of decision making units. European Journal of Operational Research.

[ref8] De Bruijn H (2002). Performance measurement in the public sector: strategies to cope with the risks of performance measurement. International Journal of Public Sector Management.

[ref9] Eddy D. M (1998). Performance measurement: problems and solutions. Health Affairs.

[ref10] Gannon B (2005). Testing for variation in technical efficiency of hospitals in Ireland. Economic and Social Review.

[ref11] Gholipour K, Delgoshai B, Masudi-Asl I, Hajinabi K, Iezadi S (2013). Comparing performance of Tabriz obstetrics and gynaecology hospitals managed as autonomous and budgetary units using Pabon Lasso method. Australian medical journal.

[ref12] Halachmi A (2002). Performance measurement: a look at some possible dysfunctions. Work Study.

[ref13] Hollingsworth B (2008). The measurement of efficiency and productivity of health care delivery. Health Economics.

[ref14] Imami-Meibodi A (2005). The principles of efficiency and productivity measurement (practical and applied) [in Persian].

[ref15] Ismail K, Thorwarth M, Arisha A (2014). Integrated decision support systems for improving emergency department performance in Irish hospitals. International Journal of Operational Research.

[ref16] Jacobs R, Smith P, Street A (2006). Measuring efficiency in health care: analytic techniques and health policy. http://dx.doi.org/10.1017/CBO9780511617492.

[ref17] Kaplan R, Norton D (2001). Transforming the Balanced Scorecard from Performance Measurement to Strategic Management: Part I. Accounting Horizons.

[ref18] Kengil B, Gökmen N, Tozan H (2010). Efficiency measures in the health services with DEA-An overview. Journal of Naval Science and Engineering.

[ref19] Kiadaliri A. A, Jafari M, Gerdtham U.-G (2013). Frontier-based techniques in measuring hospital efficiency in Iran: a systematic review and meta-regression analysis. BMC Health Services Research.

[ref20] Kirigia J. M (2013). Efficiency of Health System Units in Africa: A Data Envelopment Analysis.

[ref21] Kundurjiev T, Salchev P (2011). Technical efficiency of hospital psychiatric care in Bulgaria–assessment using Data Envelopment Analysis.

[ref22] Lynch R, Cross K (1991). Measure Up!Yardsticks for Continuous Improvement.

[ref23] Mc Kee M, Healy J (2002). Hospitals in a Changing Europe.

[ref24] Mohit A (2000). Lessons Learned in the Eastern Mediterranean Region from Integration of Mental Health Within Primary Health care in I.R. Iran.

[ref25] Najarzadeh M, Torabipoor A, Ghasemzadeh R, Salehi R (2012). Assessment of hospitals efficiency by Data Envelopment Analysis in Ahvaz in 2006-2010. Jundishapur Journal of Health Sciences.

[ref26] Neely A, Adams C, Kennerley M (2002). The performance prism: the scorecard for measuring and managing business success.

[ref27] Nekoei Moghadam M, Rooholamini M, Yazdi Feizabadi V, Hooshyar P (2012). Comparing Performance of Selected Teaching Hospitals in Kerman and Shiraz Universities of Medical Sciences, Iran, Using Pabon-Lasso Chart. Journal of Health & Development.

[ref28] Olivares-Tirado P, Tamiya N (2014). Measuring Efficiency in Special Nursing Homes in Japan: An Application of DEA Analysis. Trends and Factors in Japan’s Long-Term Care Insurance System.

[ref29] Ozcan Y (2007). Health care benchmarking and performance evaluation: an assessment using data envelopment analysis (DEA).

[ref30] Pabón Lasso H (1986). Evaluating hospital performance through simultaneous application of several indicators. Bulletin of the Pan American Health Organization (PAHO).

[ref31] Sajadi H (2011). Is there any method to compare key indicators of hospital performance simultaneously ? [in Persian]. Health Information Management.

[ref32] Shahhoseini R, Tofighi S, Jaafaripooyan E, Safiaryan R (2011). Efficiency measurement in developing countries: application of data envelopment analysis for Iranian hospitals. Health Serv Manage Res.

[ref33] Shaw C. D (2003). How can hospital performance be measured and monitored?.

[ref34] Veillard J, Guisset A.-L, Garcia-Barbero M (2004). Selection of Indicators for Hospital Performance Measurement; A report on the 3^rd^ and 4^th^ WHO Workshop.

[ref35] Zhu J (2003). Quantitative models for performance evaluation and benchmarking: data envelopment analysis with spreadsheets and DEA excel solver. http://dx.doi.org/10.1007/978-1-4757-4246-6.

